# Supplementary figures and images for: MiMeNet: Exploring microbiome-metabolome relationships using neural networks
Source: PLoS Comput Biol. 2021 May 17;17(5):e1009021. doi: 10.1371/journal.pcbi.1009021 (PMC8158931; doi:10.1371/journal.pcbi.1009021)

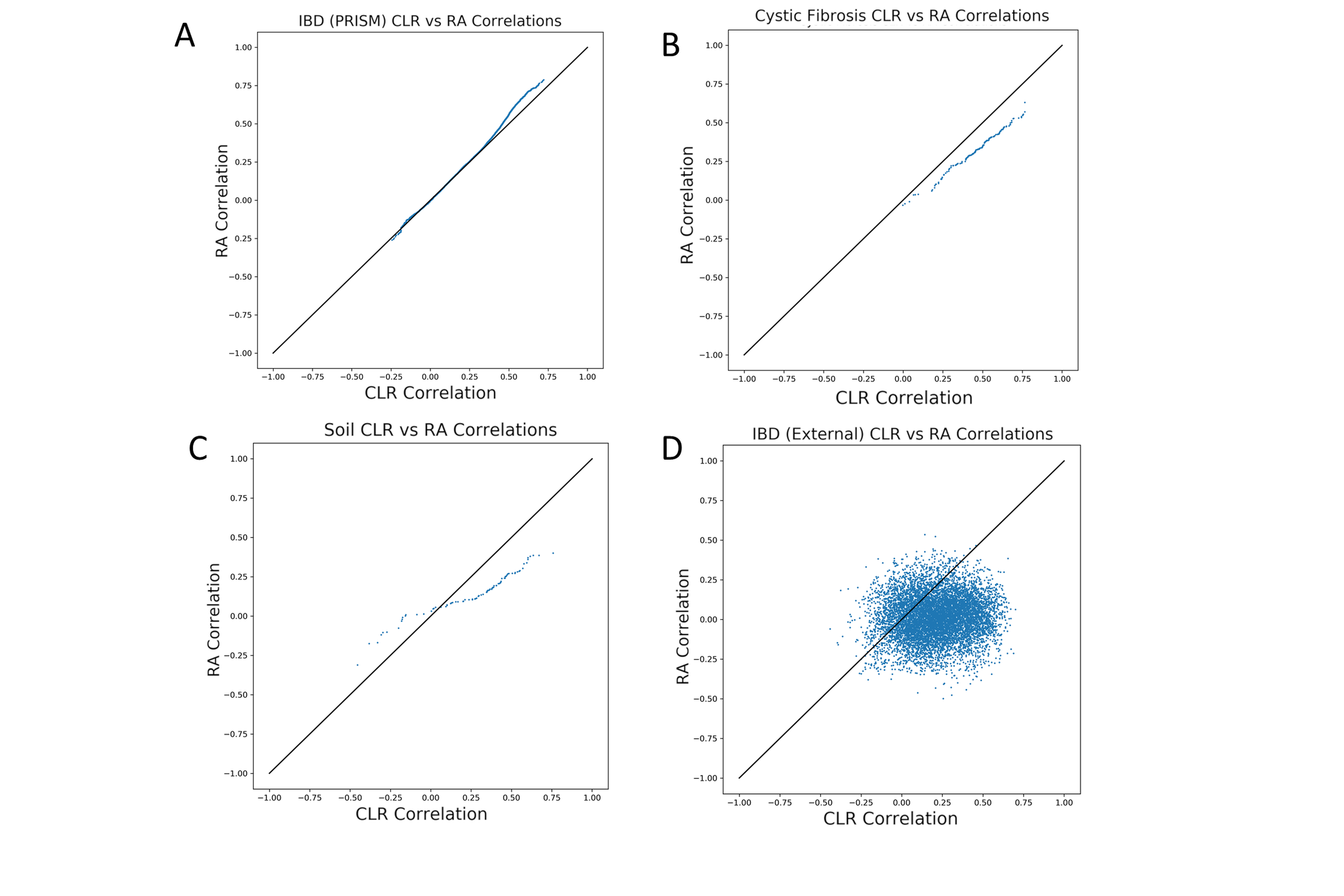

Supplement: S1 Fig — Scatterplots comparing metabolite correlation prediction between data transformed to relative abundance (RA) and centered log-ratio (CLR) for (A) IBD (PRISM), (B) cystic fibrosis, (C) soil datasets using 10 iterations of 10-fold cross-validation, and (D) IBD (External) test predictions using models trained on the full IBD (PRISM) dataset. (TIF) [file pcbi.1009021.s001.tif]

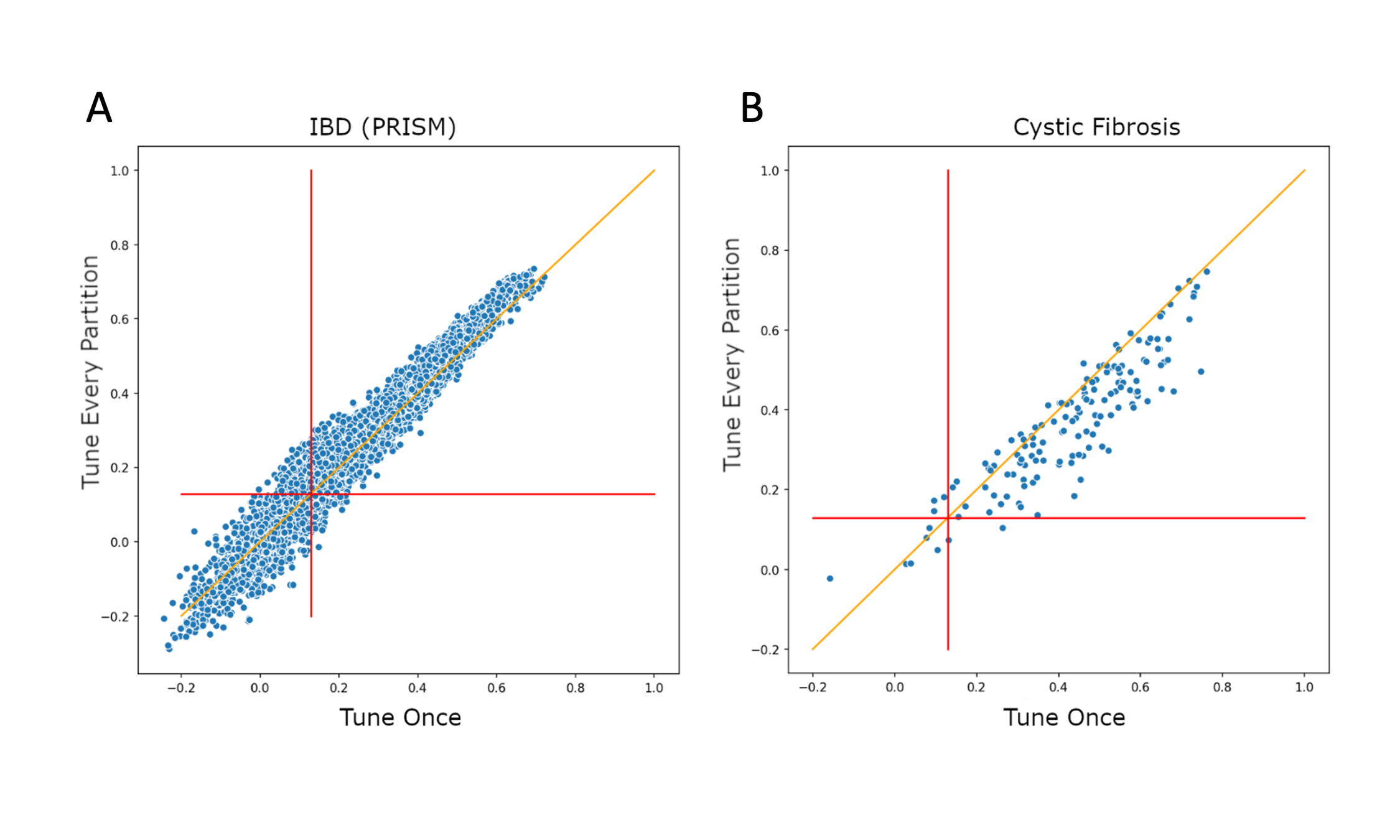

Supplement: S2 Fig — Using 10-iterations of 10-fold cross-validation, evaluations using shared hyper-parameters tuned from the first partition (Tune Once) were compared against evaluations with tuning for each partition (Tune Every Partition) for the IBD (PRISM) and cystic fibrosis dataset. Each point represents the mean SCC of a metabolite and the red lines represent the determined SCC threshold for significantly well-predicted metabolties. (TIF) [file pcbi.1009021.s002.tif]

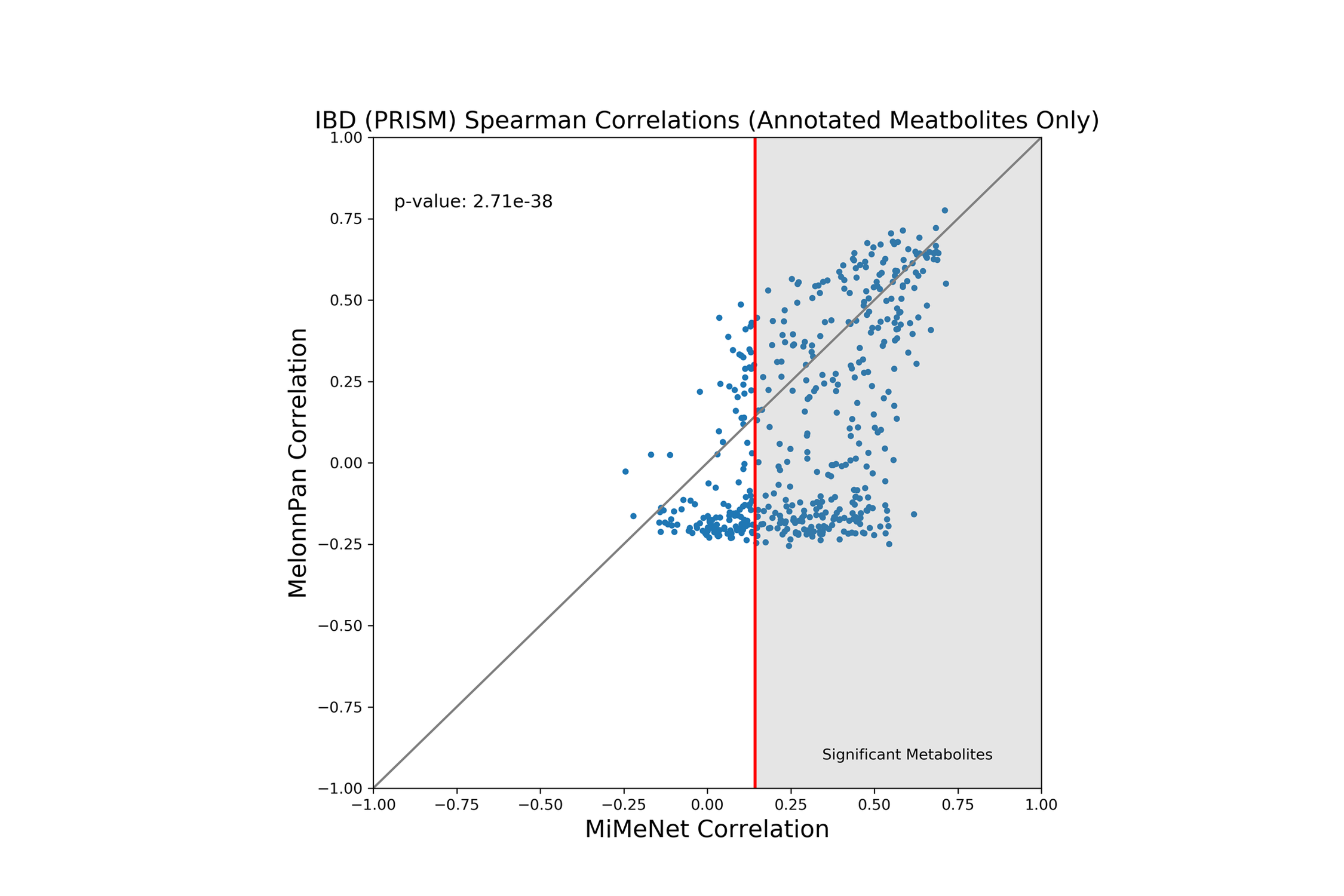

Supplement: S3 Fig — Scatterplot of mean predicted Spearman’s correlations over 10 iterations of the 10-fold cross-validation for each metabolite between MiMeNet and MelonnPan when MiMeNet is trained only on the annotated metabolites in the IBD (PRISM) dataset. (TIF) [file pcbi.1009021.s003.tif]

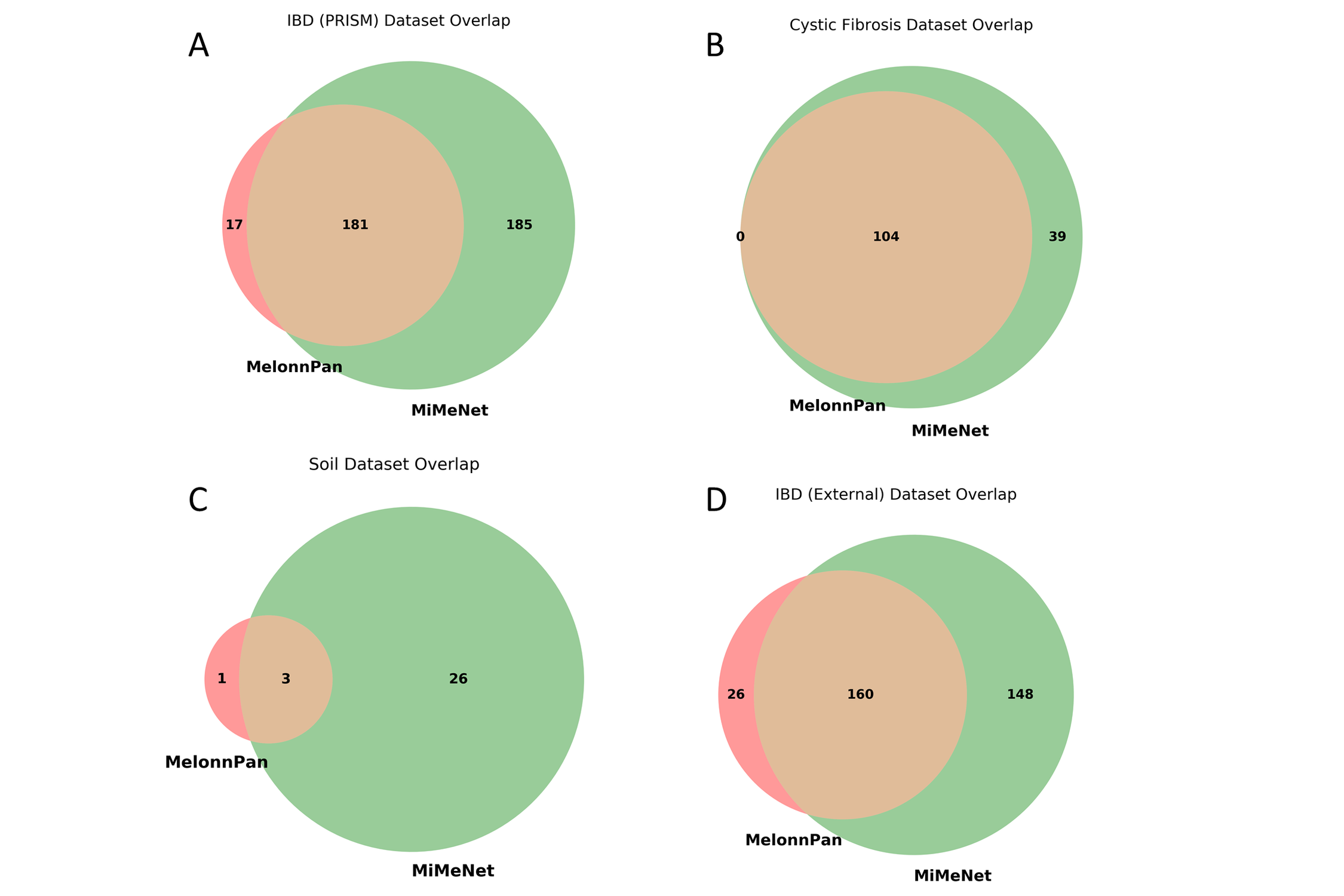

Supplement: S4 Fig — Using the correlation cutoff identified by MiMeNet, the overlap between the well-predicted metabolites is shown between MiMeNet and MelonnPan for (A) IBD (PRISM) dataset, (B) cystic fibrosis dataset, (C) and soil dataset. (D) In addition, the overlap of well-predicted metabolites is shown when training on the entire IBD (IBD) dataset and predicting on the IBD (External) dataset. (TIF) [file pcbi.1009021.s004.tif]

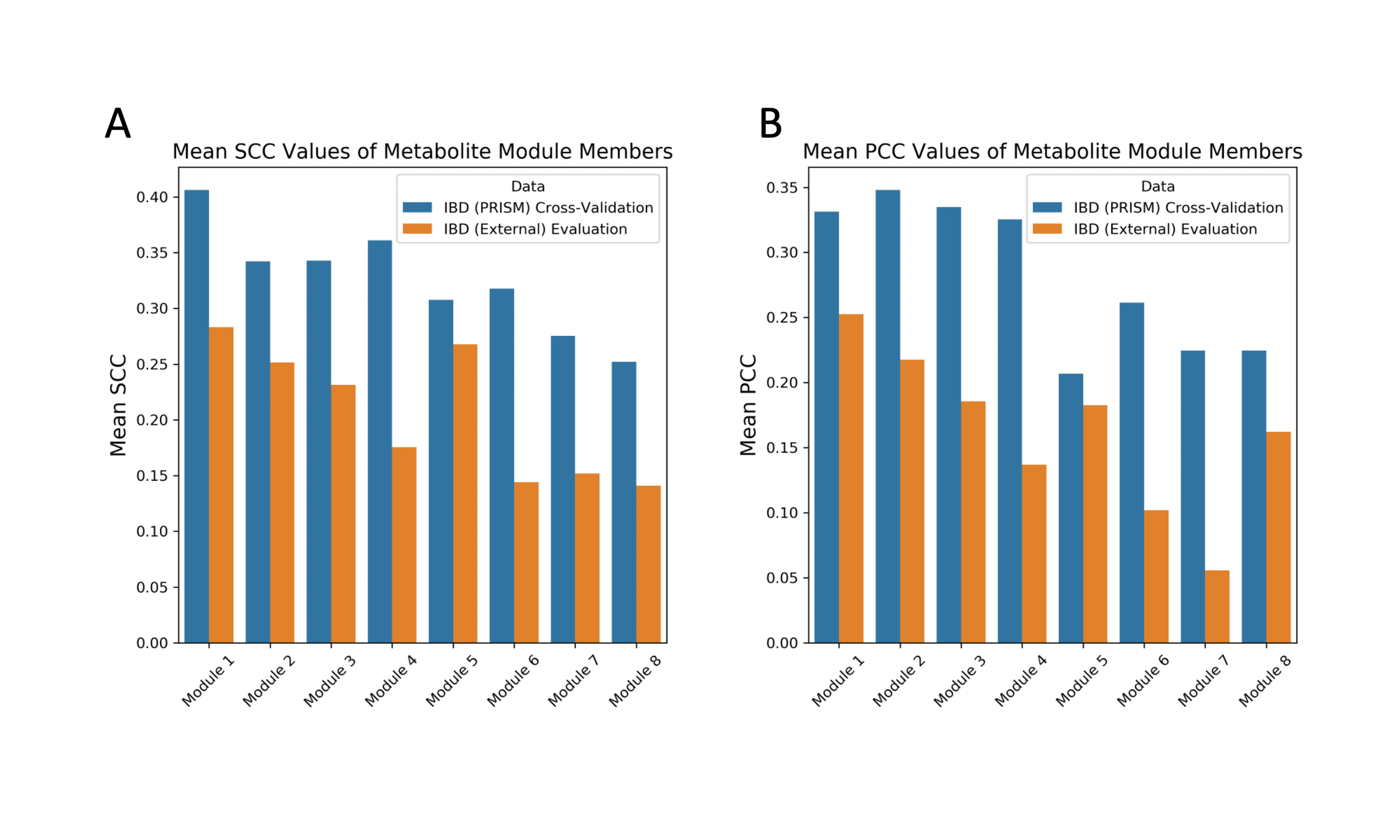

Supplement: S5 Fig — For each metabolite module, the (A) mean SCC and (B) mean PCC values of the members within the module are shown using the cross-validated evaluation on IBD (PRISM) as well as when evaluating the IBD (External) data. (TIF) [file pcbi.1009021.s005.tif]

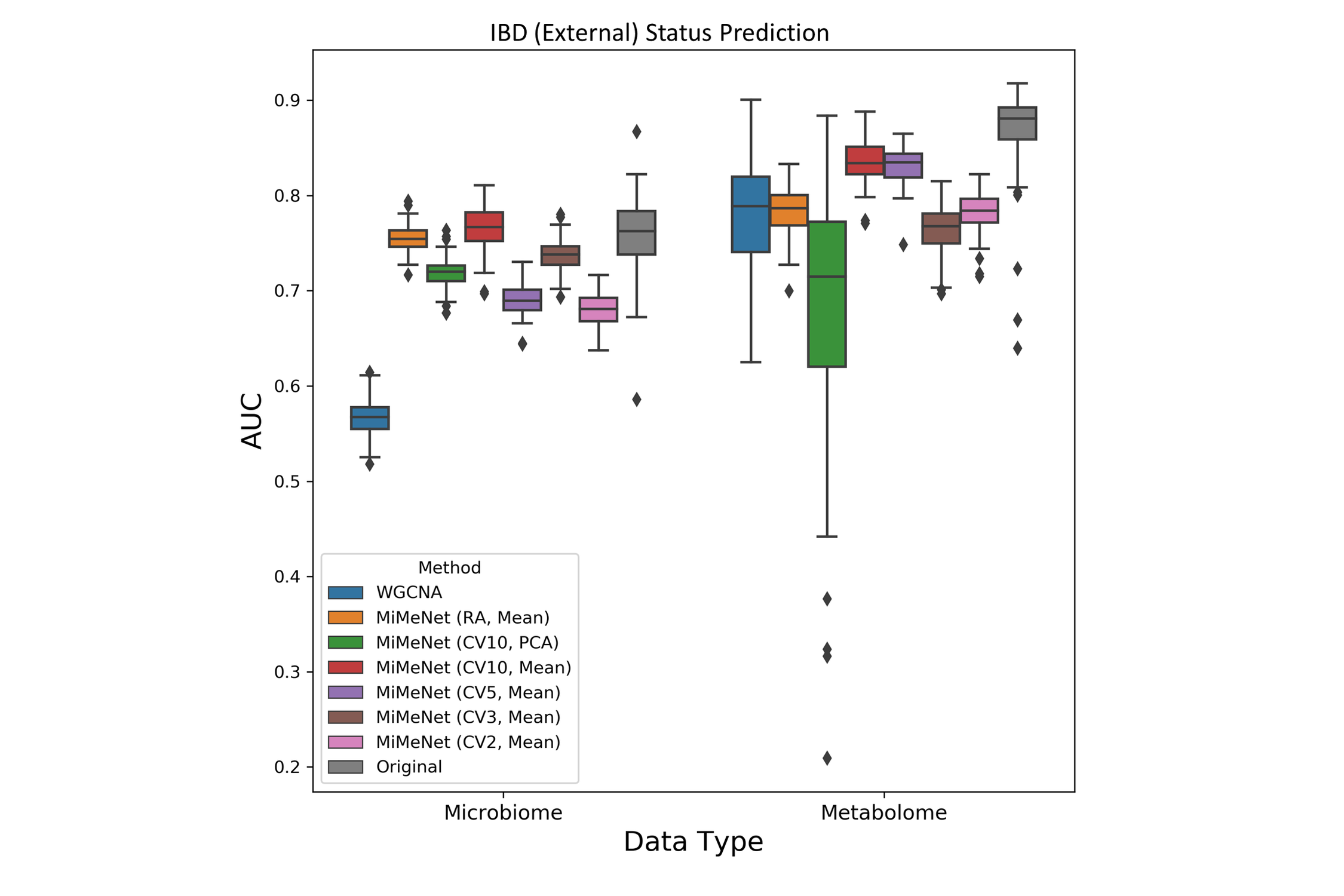

Supplement: S6 Fig — Module values were constructed using WGCNA and MiMeNet. For metabolomic modules constructed by MiMeNet, the values within the parentheses represent the compositional transformation, number of folds for cross-validation, and the aggregation method respectively. Mean aggregation calculates the mean normalized abundance value. PCA aggregation uses the first principal component of the members’ values from that module. Microbiome modules were constructed similarly with the exception that RA was always used. (TIF) [file pcbi.1009021.s006.tif]

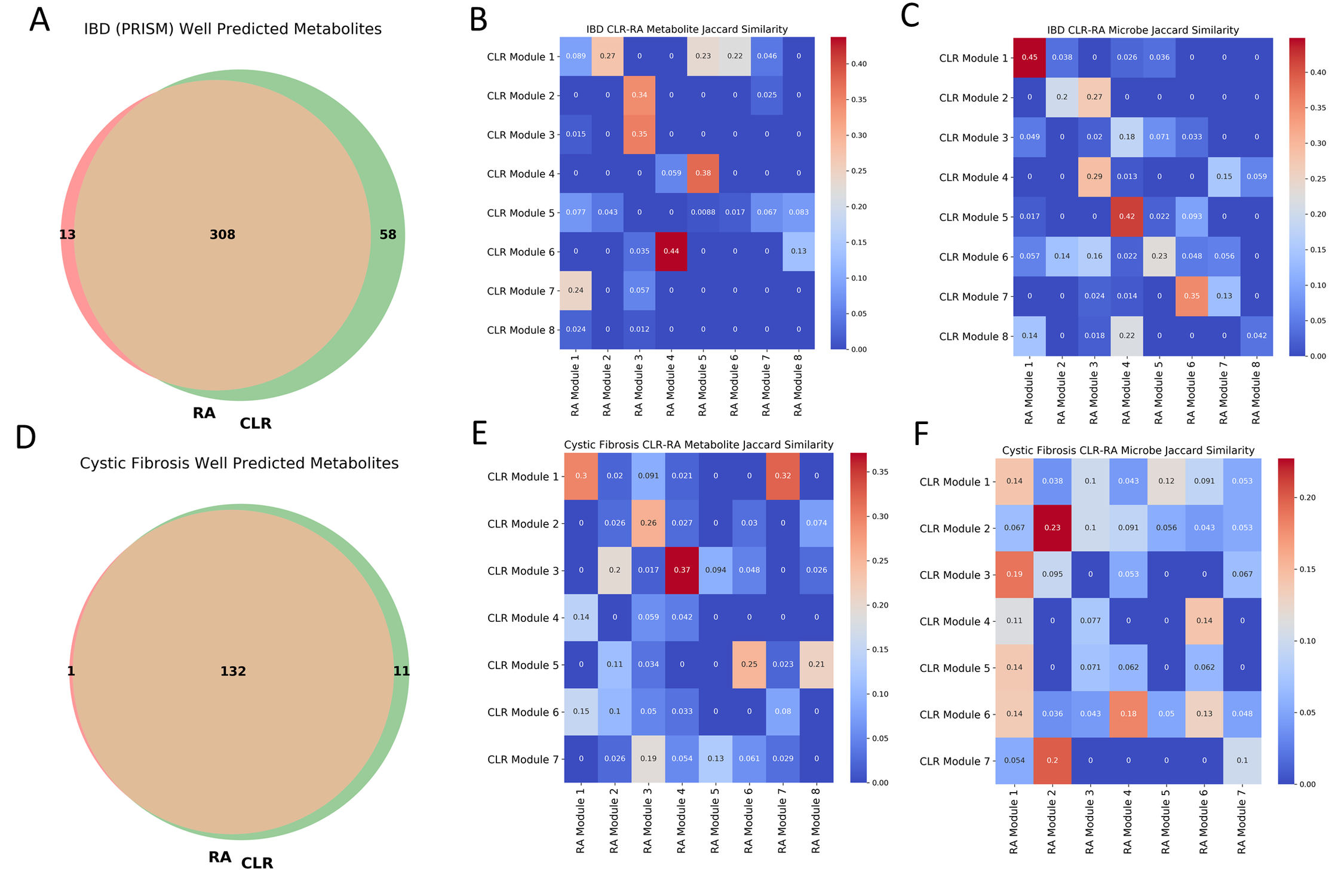

Supplement: S7 Fig — (A) Overlap of well-predicted metabolites when using relative abundance and centered log-ratio for the IBD (PRISM) dataset. Heatmaps show Jaccard similarity between membership of (B) metabolite and (C) microbial modules in the IBD (PRISM) dataset. (D) Overlap of well-predicted metabolites when using relative abundance and centered log-ratio for the cystic fibrosis dataset. Heatmaps show Jaccard similarity between membership of (E) metabolite and (F) microbial modules in the cystic fibrosis dataset. (TIF) [file pcbi.1009021.s007.tif]

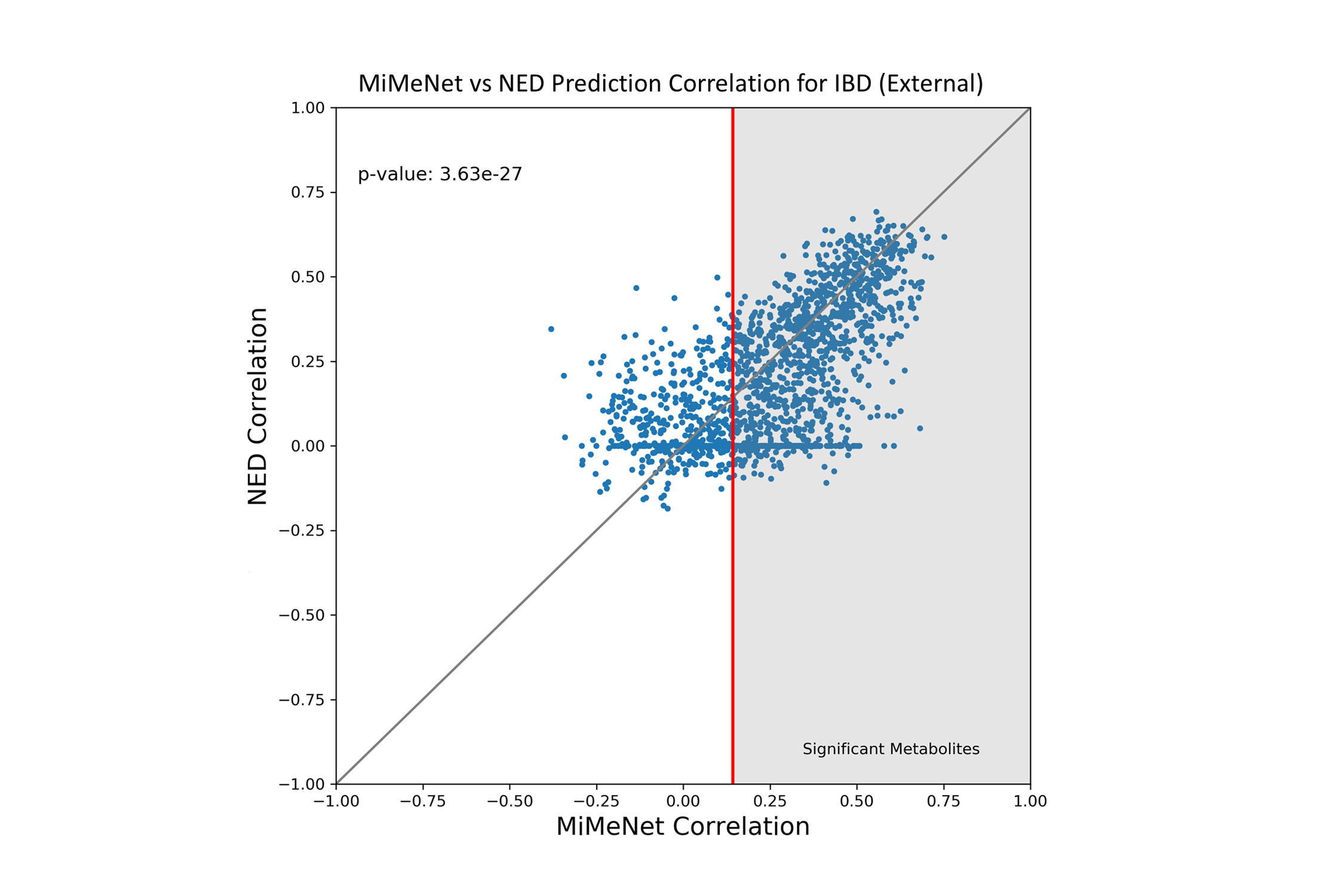

Supplement: S8 Fig — Scatterplot comparing metabolite prediction correlation between MiMeNet and NED on the IBD (External) dataset validation. The red line indicates the correlation threshold identified by MiMeNet and the gray area represents well-predicted metabolites. The one-tailed p-value (Wilcoxon sign-rank) comparing MiMeNet’s values to NED’s is shown in the top right. (TIF) [file pcbi.1009021.s008.tif]

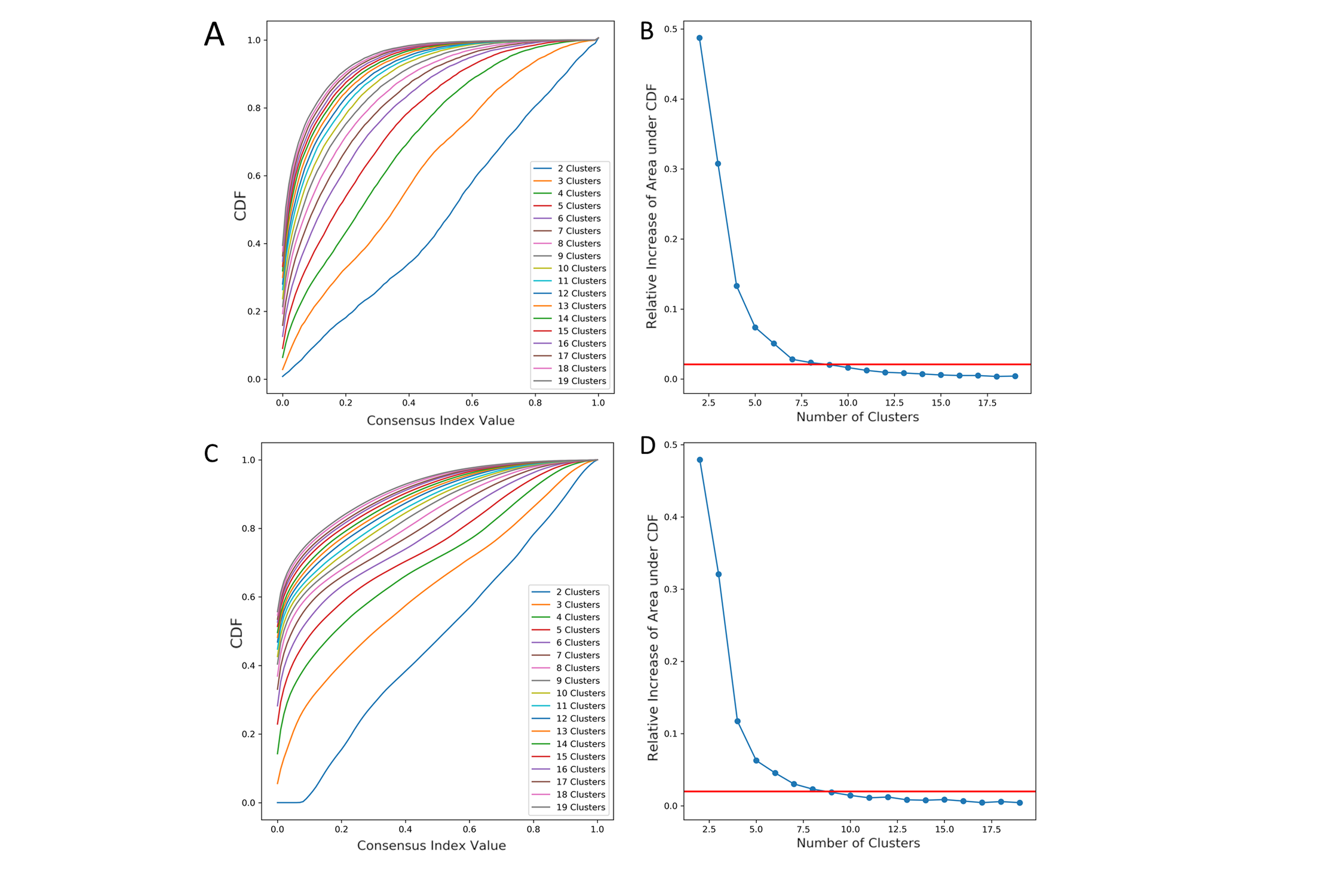

Supplement: S9 Fig — (A) The cumulative distribution functions (CDF) for varying cluster numbers and (B) change in area under the CDF is shown for clustering on the microbial features. (C) The cumulative distribution functions (CDF) for varying cluster numbers, and (D) change in area under the CDF is shown for clustering on the metabolic features. (TIF) [file pcbi.1009021.s009.tif]
